# RaCE: A rank-clustering estimation method for network meta-analysis

**DOI:** 10.1017/rsm.2025.10049

**Published:** 2025-11-13

**Authors:** Michael Pearce, Shouhao Zhou

**Affiliations:** 1 Mathematics and Statistics, Reed College, USA; 2 Public Health Sciences, Pennsylvania State University, USA

**Keywords:** item indifference, multiple comparisons, NMA, ranking, SUCRA, top cluster membership

## Abstract

Ranking multiple interventions is a crucial task in network meta-analysis (NMA) to guide clinical and policy decisions. However, conventional ranking methods often oversimplify treatment distinctions, potentially yielding misleading conclusions due to inherent uncertainty in relative intervention effects. To address these limitations, we propose a novel Bayesian rank-clustering estimation approach, termed rank-clustering estimation (RaCE), specifically developed for NMA. Rather than identifying a single “best” intervention, RaCE enables the probabilistic clustering of interventions with similar effectiveness, offering a more nuanced and parsimonious interpretation. By decoupling the clustering procedure from the NMA modeling process, RaCE is a flexible and broadly applicable approach that can accommodate different types of outcomes (binary, continuous, and survival), modeling approaches (arm-based and contrast-based), and estimation frameworks (frequentist or Bayesian). Simulation studies demonstrate that RaCE effectively captures rank-clusters even under conditions of substantial uncertainty and overlapping intervention effects, providing more reasonable result interpretation than traditional single-ranking methods. We illustrate the practical utility of RaCE through an NMA application to frontline immunochemotherapies for follicular lymphoma, revealing clinically relevant clusters among treatments previously assumed to have distinct ranks. Overall, RaCE provides a valuable tool for researchers to enhance rank estimation and interpretability, facilitating evidence-based decision-making in complex intervention landscapes.

## Highlights

- **What is already known?**
  - Network meta-analysis (NMA) is widely used to compare multiple interventions and generate treatment rankings for clinical and policy decision-making.
  - Conventional ranking reporting often oversimplifies treatment distinctions by focusing on identifying a single “best” intervention when interventions have similar effectiveness.
  - Existing methods may lead to misleading conclusions due to ranking uncertainty. For example, treatments with fewer trials tend to receive positively biased rankings, while extensively studied treatments may have negatively biased rankings.
- **What is new?**
  - Rank-clustering estimation (RaCE) for NMA is a novel Bayesian rank-clustering framework that probabilistically groups interventions with comparable effectiveness rather than imposing strict hierarchical rankings.
  - Unlike existing clustering methods embedded within specific NMA models, RaCE decouples rank-clustering from the NMA modeling process, enabling broader applicability across NMA models of different outcome types (binary, continuous, and survival), modeling approaches (arm-based and contrast-based), and estimation frameworks (frequentist and Bayesian).
- **Potential impact for RSM readers**
  - RaCE offers an advanced rank-clustering tool for NMA to enhance the interpretability of intervention comparisons, helping researchers make more informed and clinically relevant decisions.

## 1 Introduction

Network meta-analysis (NMA) is a widely adopted and powerful approach that integrates evidence from both direct and indirect comparisons, enabling simultaneous evaluation of multiple interventions within a unified model. This framework extends the scope of traditional meta-analysis to provide a more comprehensive assessment of the effectiveness of interventions. NMA is particularly valuable in settings where direct head-to-head trials are limited, offering insights into the relative rankings of treatments or interventions and informing evidence-based clinical decisions and policy development.

Despite its strengths, interpreting NMA results can be challenging, particularly when numerous interventions are involved.^1^ Reporting only the probability of being the best intervention is well recognized as potentially misleading, as it can result in erroneous conclusions especially in the presence of unequal uncertainty across the interventions being compared.^2^^,^ ^3^ Popular ranking metrics, such as the surface under the cumulative ranking curve (SUCRA), condense rank probabilities into a single numerical score that reflects the overall intervention effectiveness.^4^ While useful for summarization, SUCRA can sometimes mislead by overemphasizing rankings without accounting for clinically meaningful differences. For example, interventions with similar effect sizes may have very different SUCRA scores due to small uncertainty, potentially exaggerating distinctions that lack clinical significance.^5^ Alternatively, early methods to cluster interventions are limited to point effect estimates, which are often criticized for neglecting estimation uncertainty.^6^

To address these concerns, guidelines from Cochrane^7^ and PRISMA^8^ recommend reporting rank probabilities across all ranks rather than focusing solely on the “best” rank. Visual tools, such as rankograms and rank-heat plots, enhance the understanding of intervention performance by illustrating the distribution of rank probabilities to emphasize the uncertainty inherent in ranking results.^9^^,^ ^10^ It is also recommended that clinical researchers should focus on intervention effect sizes in addition to rankings, as a high rank does not necessarily imply a large or clinically meaningful effect size.^11^ While these approaches reduce bias and improve interpretability, they complicate the concise summarization of NMA findings, making it more challenging to distill actionable insights for clinical and policy decisions.

A compelling example of this complexity arose from a recent collaboration with the World Health Organization, where NMA was employed to evaluate the efficacy of prenatal balanced energy protein (BEP) supplements for pregnant women in low- and middle-income countries. Instead of identifying a single best BEP supplement, the priority was to determine a set of effective BEP options suitable for country- or region-specific policy decisions under varying practical constraints.^12^ This case highlights the limitations of conventional ranking approaches in NMA, motivating the investigation of NMA rank-clustering methods to align more closely with real-world decision-making needs.

Similar research questions have also attracted significant emerging interest. For example, Kong, Daly, and Béliveau^13^ introduced estimation of intervention effects with ties in contrast-based NMA models by employing generalized fused lasso to penalize all pairwise differences between treatment effects. Barrientos, Page, and Lin^14^ presented a novel Bayesian nonparametric approach with a spike and slab prior to model clustered treatment effects for binary outcomes. However, these efforts in the advanced NMA methodology predominantly embed the intervention clustering procedure directly within the NMA model itself. Subsequently, these clustering-embedded methods are highly dependent on specific NMA data structures and outcome types, limiting their methods' flexibility and generalizability.

A more versatile strategy, adopted in this work, is to *decouple* advanced rank-clustering from the NMA modeling process. Instead of embedding clustering within a predefined NMA framework, the decoupling strategy leverages the output of NMA intervention effect estimates, regardless of the underlying NMA modeling approach, as input for rank-clustering. This approach enhances applicability across various NMA estimation models, allowing researchers to select the most appropriate modeling strategy for their specific context. By avoiding constraints on model selection, this strategy broadens the applicability of rank-clustering, improves the interpretability of intervention rankings, and supports more flexible and informed decision-making. Moreover, its adaptable structure ensures compatibility with future advancements in NMA methodology, making it a robust and scalable solution for intervention ranking in complex clinical and policy settings.

Outside meta-analysis, rank-clustering methods have been rapidly developed based on ordinal comparison data when items (e.g., interventions) are inferred to be equal or indistinguishable in rank. Important early work includes Marsarotto and Varin,^15^ which developed a frequentist statistical method for estimating overall rankings of sports teams in a league that permits some teams to be equally ranked. Their method has been extended to rank-cluster additional datasets in sports^16^ and model citation exchange among academic journals.^17^ More recently, Piancastelli and Friel^18^ introduced a Bayesian statistical model for rank-clustering based on the Mallows model.^19^ Their model requires pre-specifying the number and size of rank-clusters, which was relaxed by Pearce and Erosheva^20^ in their rank-clustered Bradley–Terry–Luce (BTL) model, based on the BTL family of ranking distributions. These methods, varied in their assumptions and types of permissible data, have significantly improved accuracy in estimation, prediction, and ranking interpretability. Nevertheless, none of these approaches, despite their progress in method innovation for clustered rankings, could be directly applied to NMA as they rely on ordinal comparison data rather than continuous relative intervention effects.

In this work, we develop a general Bayesian ranking method, named *RaCE-NMA* (rank-clustering estimation for NMA, or RaCE in short), to uncover the ranking structure of intervention options in the network. Instead of identifying a single “best” intervention, RaCE-NMA identifies a cluster of interventions that are collectively ranked as best, with uncertainty, offering a practical and interpretable summary for clinical decision-making. In particular, we go beyond directly modeling the study-level NMA data for ranking clusters, but focus on clustering over NMA-estimated treatment effects. Thus, this strategy is broadly applicable to diverse existing NMA models while being compatible with future NMA modeling evolution. In practice, it can not only facilitate the decision-making for intervention selection, but also shed light on designing future head-to-head clinical comparative studies within the top rank-clustered group to strengthen or refine the existing network of evidence.

The remainder of this article is organized as follows. In Section 2, we propose the RaCE model for NMA and discuss its Bayesian estimation. Section 3 presents two simulation studies, followed by an application of the RaCE method to a real-world NMA study of 11 immunochemotherapies for follicular lymphoma.^21^ Section 5 concludes with a discussion of implications and limitations.

## 2 Rank-clustered network meta-analysis

We propose RaCE for NMA as a general Bayesian framework to uncover the ranking structure of interventions within a network. Served as a post-processing step in an NMA analysis, RaCE is a refinement method applied after conducting a standard NMA, requiring only the input of estimated means and variance–covariance matrix of intervention effects derived from any NMA model output (e.g., in Bayesian NMA, the posterior mean and variance–covariance matrix of intervention effects). In this section, we introduce notation, outline the proposed model, and describe an algorithm for computationally efficient estimation.

### 2.1 Notation

Let *J* denote the number of interventions and let 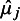 be the estimated NMA effect mean of each intervention 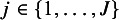 . We use 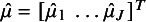 to denote the 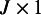 vector of estimated means. Next, let 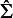 be the estimated 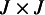 NMA variance–covariance matrix of the interventions’ effects, where 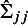 is the estimated NMA effect variance of intervention *j*, and 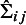 is the covariance between interventions *i* and *j*. While this definition is clear for arm-based NMA models, here we add the following specification for contrast-based NMA models: without loss of generality, we designate 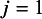 as the benchmark intervention with fixed 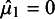 and 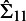 set to a small positive constant to avoid degeneracy.

The RaCE method partitions the set of interventions 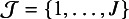 into distinct ranks. In mathematical clustering notation, a partition of an object set 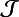 can be re-written as a collection 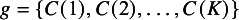 of *K* disjoint, nonempty subsets (henceforth referred to as “clusters”) of 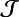 such that their union forms 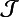 . That is, to form a complete partition of 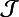 , every intervention *j* must belong to exactly one cluster and every cluster must contain at least one intervention. In our modeling, we take a probabilistic view of possible partitions of 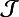 and estimate the cluster effects. Let 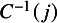 represent the cluster that contains intervention 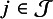 . For example, if interventions *i* and *j* belong to the k 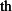 cluster, then 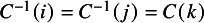 . We define 
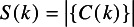
 as the size of the cluster 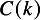 , and denote by *K* the total number of clusters in *g*. To emphasize dependence on *g*, we will write 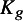 , 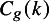 , etc. Lastly, let 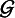 represent the collection of all possible partitions *g* of 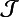 , and let 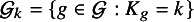 .

### 2.2 RaCE-NMA model

The proposed RaCE-NMA model may be seen as a variant of the Rank-Clustered BTL model.^20^ While the original Rank-Clustered BTL model enables inference on an overall ranking of items in which some items may be rank-clustered, it requires input data in the form of deterministic ordinal comparisons (e.g., rankings of items). In NMA rank-clustering, such input data would inappropriately neglect the ranking uncertainty. Thus, we adapt their approach for continuous data to accommodate the NMA estimation of intervention effects. Specifically, we assume the following generative Bayesian model: (2.1)

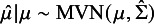


(2.2)

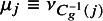


(2.3)

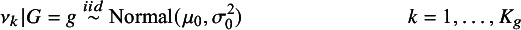


(2.4)

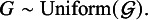

Here, we give an intuitive explanation of how the model proceeds. First, we assume the estimated NMA effect means 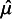 as a single data observation from a multivariate Normal 
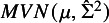
 distribution (Eq. 2.1) where 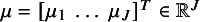 . The Multivariate Normal distribution captures covariances between the estimated intervention effects. The RaCE model employs the NMA statistics, 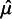 and 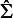 , as surrogate data for each intervention. Although in Bayesian NMA modeling, one could alternatively treat individual draws from the posterior distributions for each intervention effect as surrogate data, such a technique would artificially increase the sample size via a very large number of posterior samples. That is, posterior draws are not “data” but “results” drawn from a previous NMA model. As such, additional posterior draws do not provide additional information on the underlying treatments themselves. Instead, our choice to use the summary statistics, 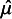 and 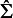 , permits a form of nonparametric cluster membership modeling in which the posterior for each 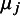 will largely mimic the posterior for the relative intervention effect of intervention *j*.

Second, the mean intervention effects 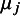 are set to equal the cluster-specific intervention effect 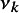 for all interventions *j* in cluster *k*. From a modeling perspective, it is equivalent to map the cluster-specific parameters 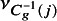 through 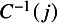 to all interventions *j* contained within the same cluster *k* (Eq. 2.2). Third, each cluster in *g* is assigned a cluster-specific effect parameter, 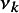 , 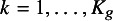 (Eq. 2.3). These parameters are drawn from a Normal prior distribution. Finally, a partition, *g*, is drawn uniformly at random from the set of all possible partitions 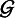 . Note that *g* is a specific partition drawn from the random variable *G*. The uniform prior aims to be uninformative, giving equal weight to all partitions^1^ (Eq. 2.4).

The model has two hyperparameters in its prior specification: The first, 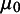 , is the prior mean on cluster-specific means. We suggest selecting 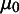 via an “objective Bayes” approach by setting 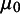 as the grand mean among intervention means, i.e., 
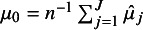
 . The second, 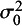 , is the prior variance on cluster-specific means. Model results may be sensitive to the choice of 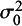 . Thus, we suggest selecting 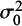 to be large, inducing an uninformative prior. One reasonable choice is to set 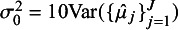 .

Furthermore, the model contains two sets of parameters to be estimated. The partition parameter, *g*, represents the (unordered) clustering structure among the interventions. The cluster-specific parameters, 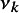 represent the estimated average intervention effect among interventions in cluster *k*. Given 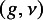 , we can rank each intervention from most to least effective by simply ordering the values in 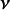 and subsequently determining which interventions belong to each cluster according to *g*. Thus, the RaCE model induces *rank-clusters* among interventions.

### 2.3 Rank-clustering interpretation

The RaCE approach represents a fundamental departure from conventional NMA ranking methods in both the construction and interpretation of treatment rankings. Conventional NMA ranking metrics, such as SUCRA, were built upon conventional posterior rank probabilities derived through exhaustive pairwise comparisons that assume strict orderings and do not permit ties. As a result, interventions that are statistically indistinguishable in terms of treatment effect are still forced into a strict rank hierarchy, which can further lead to overstated rank differences between interventions with similar effectiveness.

Moreover, methods based on conventional rank probabilities, including SUCRA, are known to be sensitive to estimation variance. Interventions with limited supporting data or higher uncertainty can attain disproportionately favorable rankings due to tail mass. This phenomenon is demonstrated in Simulation Study 2 (Section 3.2), where treatment 4, characterized by large uncertainty, was spuriously ranked above more consistently supported interventions such as treatment 1 with a notable probability.

In contrast, RaCE mitigates these limitations by employing a probabilistic clustering framework for treatment ranking. Rather than assigning a fixed, strictly ordered rank to each intervention, RaCE introduces a latent partition structure in which treatments with similar estimated effects are grouped into the same rank cluster. This framework jointly estimates both the number of rank clusters and the assignment of treatments to clusters, while explicitly accounting for uncertainty in treatment effect estimates. Each cluster is associated with a latent effect, and all treatments within a cluster are considered indistinguishable in terms of intervention effect and share the same rank.

To translate clusters into numeric rankings, we adopt the convention of assigning each treatment the lowest (i.e., most favorable) rank among its cluster. For example, if the partition structure is given by 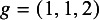 and the corresponding cluster-level effects are 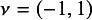 (assuming smaller 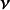 indicates better treatment effect), then treatments 1 and 2 are grouped in the top cluster and assigned rank 1, while treatment 3 is assigned rank 3. Notably, no treatment is assigned rank 2 under this convention.

It is important to clarify that in RaCE, the posterior rank probabilities are defined based on the latent clustering structure, and thus differ both conceptually and empirically from conventional rank probabilities. Specifically, the posterior rank probabilities for each intervention still sum to one across all possible ranks, but the probabilities for a given rank do not necessarily sum to one across interventions, due to the possibility of ties. For interpretability of NMA ranking results, investigators may focus on identifying interventions with a high posterior probability of belonging to the top-ranked cluster. See Section 4 for an illustration of this interpretation in a real-world application. For notational clarity, we refer to these RaCE-derived probabilities as “posterior rank-clustering probabilities” to distinguish them from conventional posterior rank probabilities used in SUCRA and related methods.

### 2.4 Bayesian estimation

Bayesian models can be slow and computationally expensive to estimate. As such, it is important to develop an efficient estimation algorithm. Here, we propose a fast estimation algorithm for the Bayesian RaCE-NMA model based on a Gibbs sampler.

The model contains two parameters, *g* and 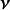 . We estimate *g* and 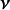 using a reversible jump Markov chain Monte Carlo (RJMCMC) Gibbs sampler that alternates between updating *g* and each 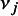 via their full conditionals. The sampler is summarized in Algorithm 1.

**Figure:**
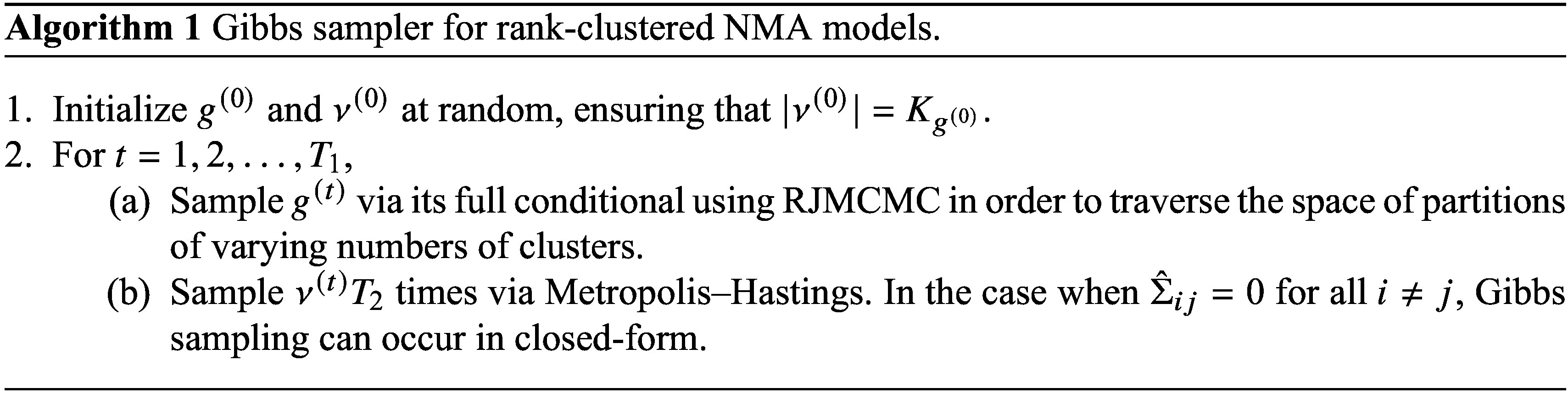

Steps 2(a) and 2(b) proceed similarly to a related algorithm for the Rank-Clustered BTL model. In Step 2(a), a new cluster structure *g* is proposed by either splitting an existing cluster in two (a “birth” in the terminology of Green^22^) or combining two existing adjacent clusters into one (“death”). This procedure permits efficient exploration of the space of possible partitions, which may be extremely large. In Step 2(b), we iteratively sample each 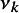 , 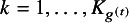 via Metropolis–Hastings. As noted before, when 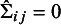 for all 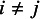 , Gibbs sampling can occur in closed form due to the conjugacy of the Normal prior placed on each 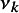 with the assumed Normal data model. Further details of the algorithm are sufficiently technical and details are thus relegated to Appendix A.

We provide a few practical notes on model estimation. The number of outer Gibbs steps, 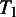 , should be sufficiently large to allow for convergence of the MCMC chain. Specific choices are context-dependent, but often at least 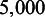 steps are needed. Step 2(a) performs RJMCMC on clusters of objects. Since RJMCMC can be slow to converge in high dimensions, it is important to run multiple chains and assess for mixing and convergence.^23^ Step 2(b) relies on Metropolis–Hastings, which may not be efficient. Thus, we find 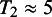 is appropriate for posterior sampling.

## 3 Simulation studies

We conduct two simulation studies to demonstrate properties of the proposed model. In the first, we explore how the model estimates rank-clusters among interventions as the variance and degree of separation among their posterior distributions changes. In the second, we demonstrate rank-clustering in the regime when the relative intervention effect is highly uncertain for a specific intervention.

### 3.1 Study 1: Rank-clustering by degree of separation

In our first simulation study, we assess the accuracy of the proposed RaCE model in estimating rank-clusters of interventions whose posterior distributions of relative intervention effects are either equal or not. We refer to pairs of interventions with different true effect parameters as “*distinct*,” and use “*separation*” to describe the degree of overlap (or lack thereof) in their posterior distributions. This distinction is critical: interventions can be truly distinct in effect yet poorly separated due to high uncertainty. By varying the degree of separation among the posterior distributions of relative intervention effects through various scenarios, we evaluate RaCE model’s performance to recover the correct clustering structure.

**Figure 1:**
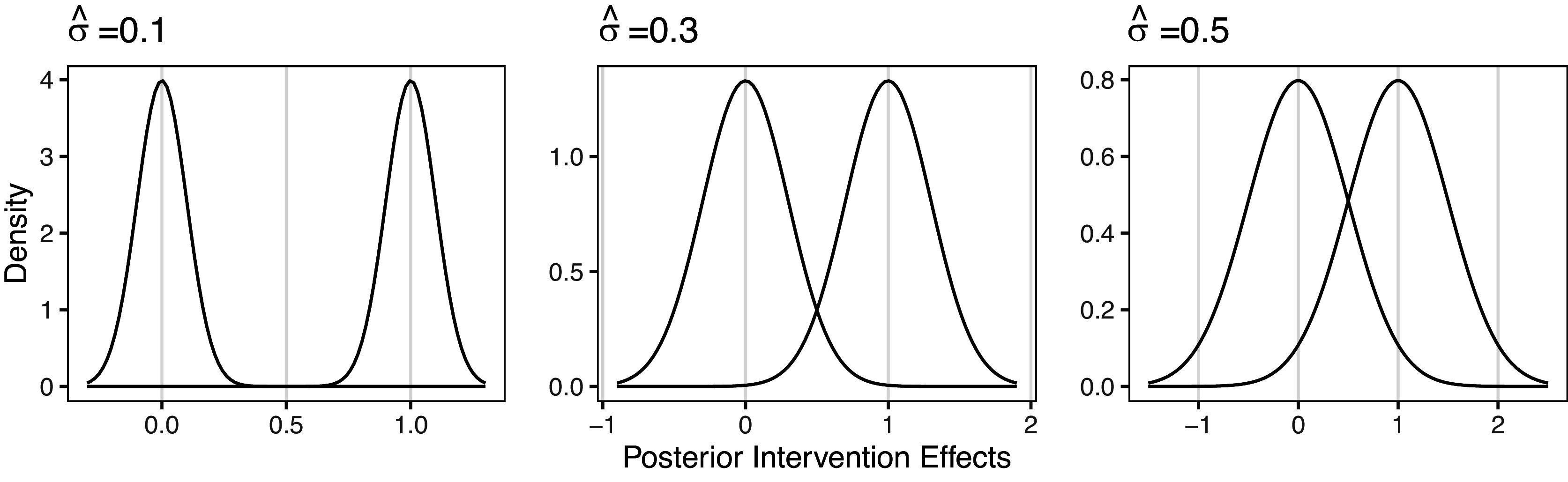
Effect of 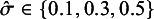 on the degree of separation between two posterior distributions with distinct means, 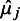 , 1 unit apart.

Specifically, we consider analyses with 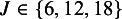 interventions, which are truly rank-clustered into 
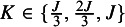
 clusters. Note that when 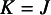 , all interventions are distinct. We randomly assign each intervention a posterior mean, 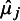 , to a value in the set 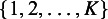 , assuming it is identical to the true mean effect. During random assignment, we also ensure each value contains at least one intervention so that there are *K* clusters, each one unit apart in mean. Then, we set the posterior standard deviation of each treatment to 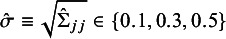 , where every intervention *j* has the same value for 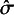 . For simplicity, we set covariances 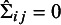 . The effect of each value of 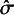 is shown in Figure 1. It can be seen that when 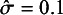 , interventions have clearly separated posterior intervention effects. When 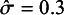 , interventions are distinct but with some overlap. When 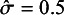 interventions are again distinct but exhibit substantial overlap. Furthermore, as 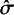 grows, there is greater uncertainty in each treatment effect’s posterior distribution. As such, we should expect less certainty in rank-clustering treatments with large 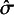 , even when they have the same estimated mean, 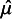 .

**Figure 2:**
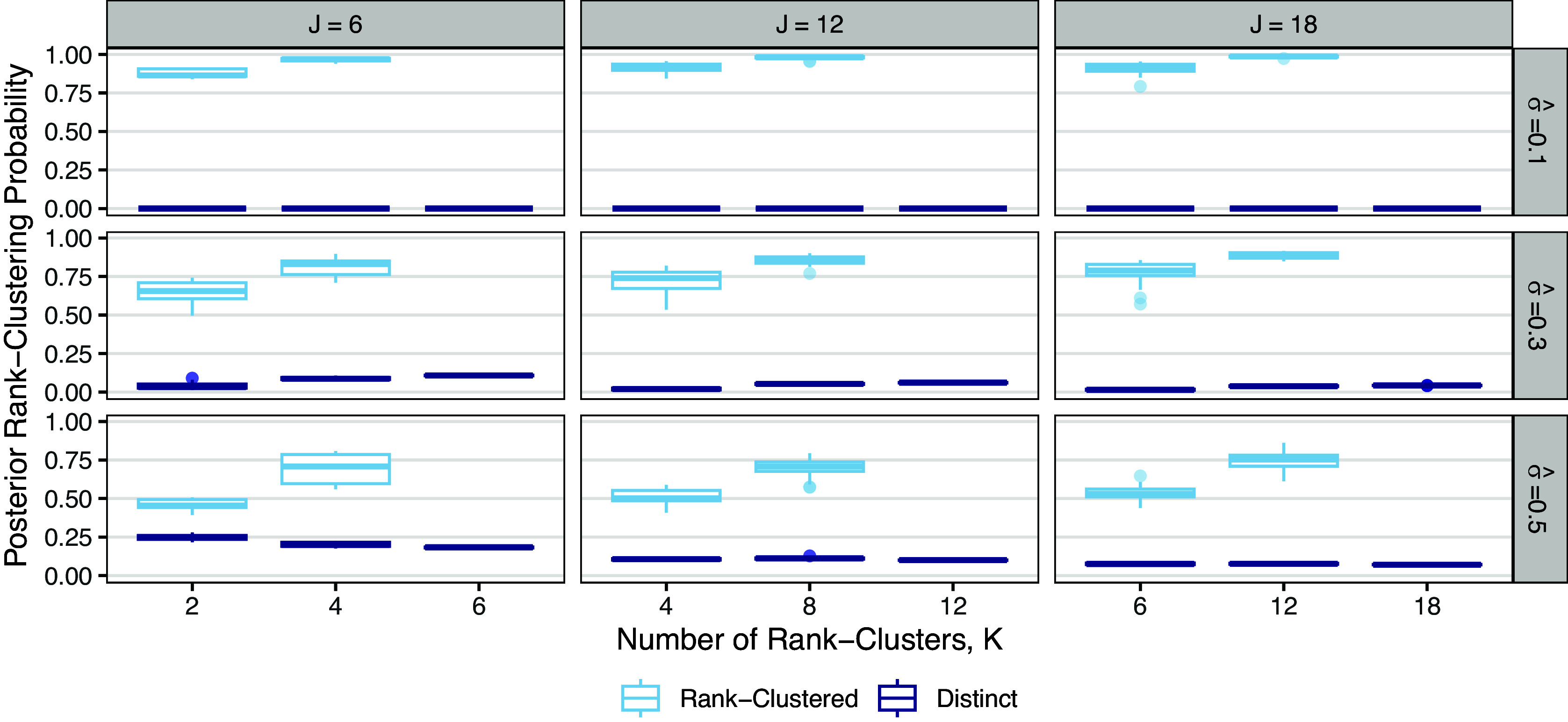
Posterior rank-clustering probability for rank-clustered and distinct pairs of interventions across varying numbers of interventions, *J*, numbers of rank-clusters, *K*, and their relative separation in average intervention effect, 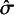 .

Finally, for each combination of *J*, *K*, and 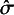 , we run 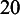 independent simulations to vary the specific rank-clustering structure. In each, we record the posterior rank-clustering probability for across pairs of interventions which are rank-clustered or distinct. Results are shown in Figure 2.

Figure 2 shows that the RaCE model appropriately assigns high probabilities of rank-clustering to interventions with equal means (light blue) and low probabilities of rank-clustering to interventions with distinct means (dark blue). This indicates accurate estimation of rank-clustering. For fixed 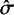 , we observe similar performance as *J* varies. For fixed *J* and 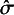 , estimation accuracy generally increases as *K* increases (i.e., higher rank-clustering probabilities for rank-clustered treatments and lower rank-clustering probabilities for treatments with distinct means), indicating that the model performs better when there are fewer sets of interventions with equal means (i.e., more clusters). Regarding 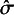 , the model assigns low posterior probability to rank-clustering interventions with distinct means when 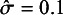 or 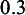 . This is a direct result of the clear separation of the posterior distributions (see Figure 1). Alternatively when 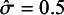 , posterior distributions significantly overlap and posterior probabilities of rank-clustering interventions with distinct means are slightly higher. As 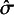 increases, the model rank-clusters interventions with equal means with probability as low as 0.50. As such, the model is somewhat conservative in clustering interventions. This conservatism may be seen as appropriate given that the intervention effects are highly uncertain for large 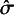 .

### 3.2 Study 2: Highly uncertain intervention effect

**Figure 3:**
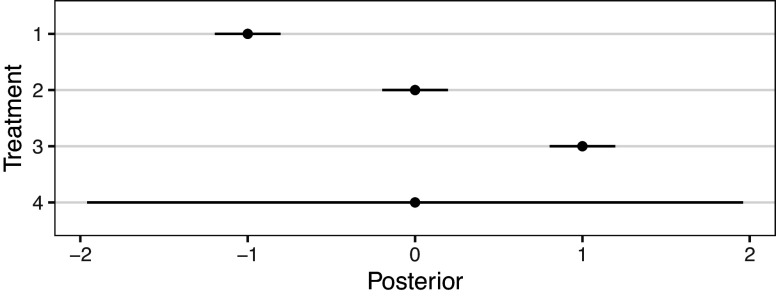
Point estimates and 95% credible intervals of assumed posterior distributions of the relative intervention effect for each intervention 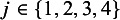 .

In our second simulation study, we assess the ability of the model to infer rank-clusters in the presence of an intervention whose relative effect is highly uncertain relative to the rest. Specifically, we consider the case when there are 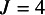 interventions, with posterior means 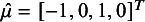 , standard deviations 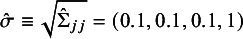 , and covariances 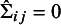 . By convention, small values for relative effects indicate a more efficacious treatment. A forest plot of the assumed posterior distributions of each intervention, as might be obtained from a traditional NMA, is shown in Figure 3. As can be seen, interventions 1–3 are distinct in relative intervention effect and well-separated in distribution, while intervention 4 is highly uncertain and has a posterior distribution that overlaps substantially with all other interventions.

**Table 1:** Posterior probability that each pair of interventions is rank-clustered

	2	3	4
1	0	0	0.18
2		0	0.40
3			0.19

We apply the RaCE model to this hypothetical dataset. Table 1 displays the estimated posterior probability that each pair of interventions is rank-clustered. We observe that interventions 1–3 have no posterior probability of rank-clustering among themselves. However, intervention 4 has a combined 0.77 probability of rank-clustering with the remaining interventions. The largest probability (0.40) belongs to intervention 2, with whom it shares a mean intervention effect. The modest probability of rank-clustering may be attributed to the considerable uncertainty regarding the efficacy of treatment 4. To a lesser but still meaningful extent, intervention 4 is rank-clustered with each of interventions 1 and 3, reflecting the overlapping nature of their posterior distributions and thus reasonable uncertainty regarding the rank-clustering structure. Furthermore, the model assigns the remaining 0.23 probability that intervention 4 is distinct from the remaining interventions.

**Figure 4:**
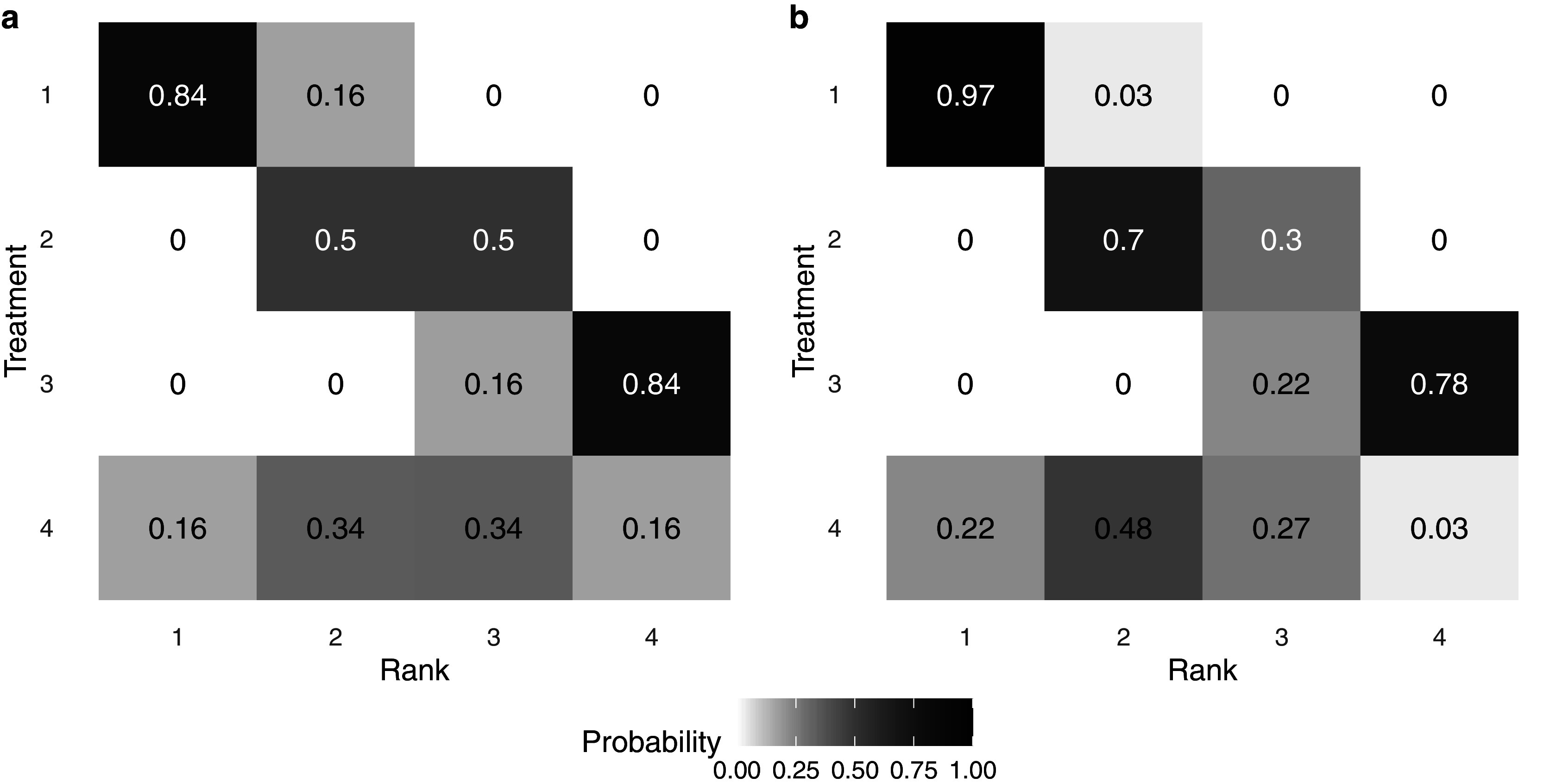
Posterior probability of each intervention belonging to each rank level under a traditional analysis (a) and the proposed RaCE model (b).

Finally, Figure 4 displays a heat map of the posterior probability that each intervention is assigned each rank level, under two distinct methods of analysis. The color and number indicate a model-specific posterior probability that a treatment (on the *y*-axis) is assigned a given rank (on the *x*-axis); darker colors indicate a higher posterior probability. Panel (a) displays results under the traditional assumption that each intervention must be assigned a distinct rank. Alternatively, panel (b) displays results under the proposed RaCE model. Note that in panel (a), the probabilities in each row and column sum to 1 due to the assumption that each intervention requires a unique rank. In contrast, in panel (b), only row probabilities sum to 1, a result of the RaCE model permitting rank-clusters.

In the traditional analysis (panel (a)), the substantial uncertainty associated with Treatment 4 results in a lower probability that treatment 1 is ranked in first place. In contrast, the RaCE model (panel (b)) assigns treatment 1 a 0.97 posterior probability of belonging to first-place cluster, substantially less affected by the inclusion of the uncertain treatment 4 in the comparison. At the same time, RaCE permits treatment 4 to simultaneously belong to first-place cluster with a posterior probability of 0.22. These results illustrate how RaCE distinguishes high-certainty evidence from high-uncertainty estimates: it confidently identifies treatment 1 as belonging to the “best” class, while still accommodating some probability that treatment 4 may be comparably or more effective.

## 4 Case study

**Figure 5:**
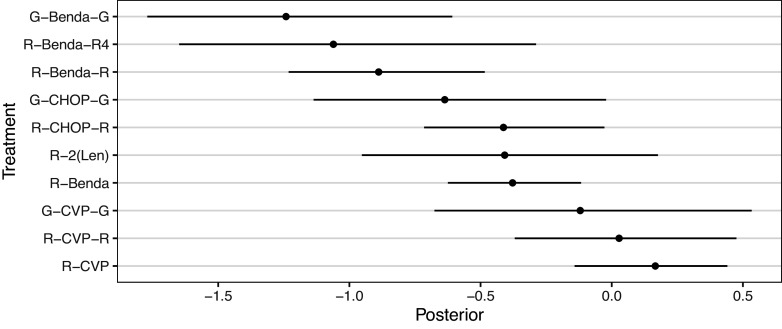
Forest plot replicating the posterior distributions of the relative treatment effects of 11 front-line immunochemotherapies for follicular lymphoma.^21^ Point estimates and 95% credible intervals are shown. R-CHOP has a relative treatment effect of 0 with no uncertainty by construction as the baseline treatment. A smaller value indicates a more effective treatment.

We demonstrate the RaCE-NMA methodology on the results of a real NMA.^21^ The study compares the efficacy of 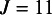 front-line immunochemotherapies for follicular lymphoma. A forest plot of the posterior distributions of the relative treatment effect^2^ for each of the 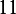 treatments is displayed in Figure 5. We note that Wang et al.^21^ treat the previous standard of care, *R-CHOP*, as the baseline. Thus, its posterior relative intervention effect is a point-mass at 0 by construction. Wang et al.^21^ found a 72% posterior probability that G-Benda-G is the best (i.e., most effective) treatment among the 11 compared; R-Benda-R4 and R-Benda-R have 25% and 3% posterior probabilities, respectively, of being the best treatment. For each of these three treatments, a posterior 95% credible interval for their rank includes 1, indicating some evidence the treatment is in first place. The study calculated posterior probabilities and credible intervals by ordering treatments by their posterior estimates of relative treatment effects in each posterior draw to produce a posterior draw for the ranking of treatments, and then calculating marginal probabilities based on rankings.

We observe that in Figure 5 the posterior distributions of the relative treatment effects for each treatment are highly overlapping. Thus, the assumption made by Wang et al.^21^ that each treatment receives a unique rank is potentially inappropriate. That is, some treatments may be equally effective at treating follicular lymphoma, or simply indistinguishable in efficacy, and thus rank-clustered. Therefore, we apply the RaCE-NMA model to examine if certain treatments are rank-clustered for first-place.

We fit the RaCE-NMA model to the results of Wang et al.^21^ post-hoc. Specifically, we input as data the posterior means, 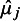 for each treatment *j* and variance–covariance matrix 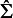 . Since R-CHOP ( 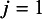 ) is the baseline treatment, there is artificial certainty in its relative treatment effect in the form of a point-mass posterior at 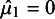 . To ensure a proper posterior distribution in the RaCE-NMA model, we thus set 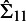 to a comparatively small positive value, specifically, 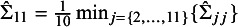 and set 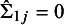 , 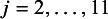 . We set minimally informative hyperparameters of 
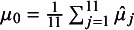
 and 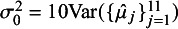 . We ran four independent chains, each with 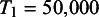 and 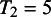 and removed the first half of each as burn-in. See Appendix B for trace plots, which demonstrate good mixing and convergence.

**Figure 6:**
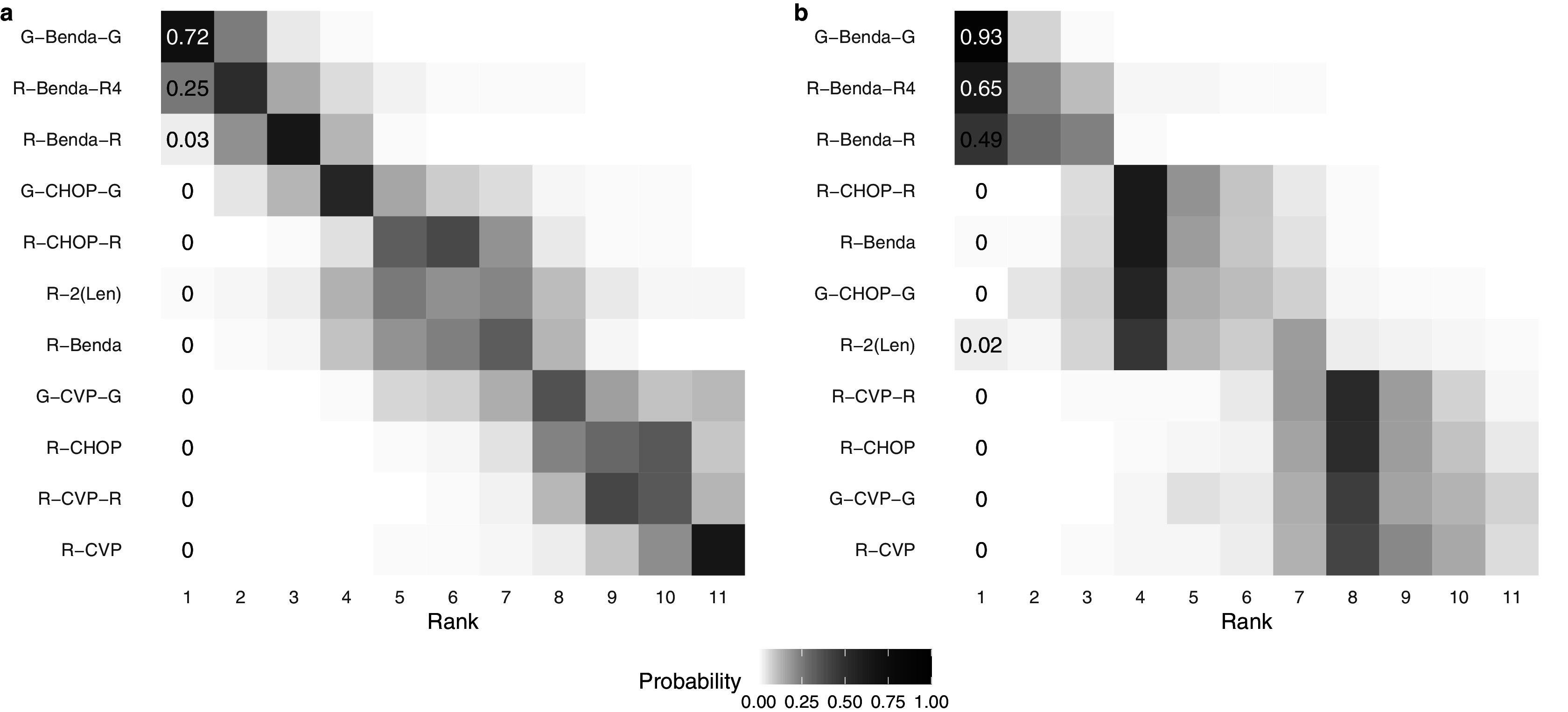
Posterior probability of each treatment belonging to each rank level under the NMA model of Wang et al.^21^ (a) and the proposed RaCE model (b).

Figure 6 displays heat maps of the estimated ranks of each treatment according to the original analysis by Wang et al.^21^ (a) and the proposed RaCE model (b). As in Figure 4, the color and number indicate a model-specific posterior probability that a treatment (on the *y*-axis) is assigned a given rank (on the *x*-axis); darker colors indicate a higher posterior probability. In panel (a), the probabilities in each row and column sum to 1 due to the assumption that each treatment requires a unique rank. In contrast, in panel (b), only row probabilities sum to 1, a result of the RaCE model permitting rank-clusters.

According to the RaCE model, G-Benda-G, R-Benda-R4, and R-Benda-R all have substantial posterior rank-clustering probability of belonging to first place. Hence, the model suggests that these treatments form a cluster of treatments that are simultaneously the most effective. By comparison, the original analysis estimates a small or zero posterior probability of first place for each treatment after G-Benda-G, a result of the modeling assumption of distinct ranks. Although not of primary interest to this analysis, we visually observe a rank-cluster of treatments R-CHOP-R, R-Benda, G-CHOP-G, and R-2(Len) behind the initial rank-cluster, and a “last place” rank-cluster of treatments R-CVP-R, R-CHOP, G-CVP-G, and R-CVP.^3^

**Figure 7:**
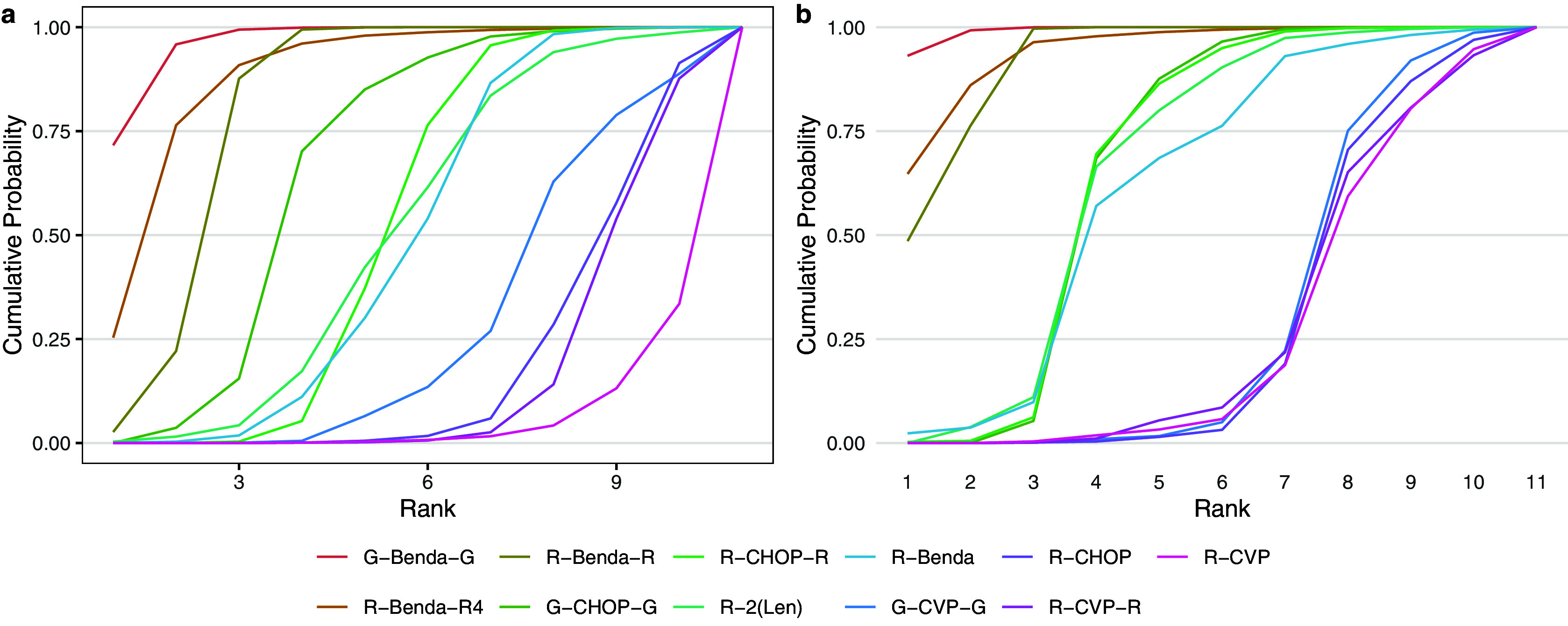
Cumulative ranking probability of each treatment under the NMA model of Wang et al.^21^ (a) and the proposed rank-clustering model (b).

Second, Figure 7 displays the cumulative posterior probability of each treatment belonging to each rank level under the original analysis of Wang et al.^21^ (a) and our proposed rank-clustering analysis (b).

The cumulative probabilities under the RaCE-NMA model more quickly increase to 1. This result is explained by rank-clustering in the proposed model, thus permitting interventions to share lower-numbered ranks in addition to the clustering effect.

Third, Table 2 displays the SUCRA and the median number of “strictly better” treatments for each model. For the purpose of illustration, we extend the concept of SUCRA^4^ for the RaCE model based on the cumulative rank-clustering probabilities: a value of 1 indicates a treatment is certain to be in the “best” class, while a value of 0 indicates a treatment is certain to be the worst. The median number of “strictly better” treatments is calculated by counting the number of treatments in each posterior draw with a strictly better ranking than any given treatment and subsequently taking the median number among the posterior draws.

**Table 2:** SUCRA and median number of better treatments (MNBT) corresponding to each treatment under original analysis by Wang et al.^21^ and under the proposed RaCE model

	SUCRA		MNBT (50% CI)	
Treatment	(Wang et al.)	(RaCE model)	(Wang et al.)	(RaCE model)
G-Benda-G	0.967	0.992	0 (0, 1)	0 (0, 0)
R-Benda-R4	0.884	0.943	1 (0, 1)	0 (0, 1)
R-Benda-R	0.812	0.925	2 (2, 2)	1 (0, 1)
G-CHOP-G	0.663	0.647	3 (3, 4)	3 (3, 4)
R-CHOP-R	0.514	0.657	5 (4, 5)	3 (3, 4)
R-2(Len)	0.501	0.604	5 (4, 6)	3 (3, 5)
R-Benda	0.482	0.657	5 (4, 6)	3 (3, 4)
G-CVP-G	0.278	0.276	7 (6, 8)	7 (7, 8)
R-CHOP	0.186	0.279	8 (7, 9)	7 (7, 8)
R-CVP-R	0.159	0.296	8 (8, 9)	7 (7, 7)
R-CVP	0.054	0.264	10 (9, 10)	7 (7, 8)

For the former, we observe that the RaCE-SUCRA is generally higher for each treatment to accommodate possible ties in ranking. For the latter, we see that three treatments credibly have 0 treatments strictly better than them. As a result, we have reasonable evidence that G-Benda-G, R-Benda-R4, and R-Benda-R are rank-clustered in first place, representing a class of three “best” treatments.

## 5 Discussion

This study introduces a novel rank-clustering estimation approach, RaCE, designed to enhance the interpretability and applicability of intervention rankings in NMA. Unlike conventional ranking methods that focus on identifying a single “best” intervention, RaCE systematically clusters interventions with similar effectiveness while automatically accommodating the uncertainty in intervention effect estimates. By shifting the focus from absolute rankings to probabilistic clustering, RaCE provides a more robust and meaningful summary of comparative performance, reducing the risk of overstating minor differences between interventions.

RaCE offers several key advantages. First, RaCE provides a structured way to assess intervention equality, addressing a critical limitation of conventional NMA ranking methods that may lead to misleading conclusions in clinical decision-making.^2^^,^ ^24^ In many real-world applications, identifying a set of top-performing interventions is more relevant than selecting a single best intervention.^14^ By clustering interventions with similar effectiveness, RaCE helps distinguish truly superior interventions from those that are statistically indistinguishable, improving the reliability of results interpretation. This shift from strict ranking to rank-clustering provides a more reliable framework for evaluating intervention equivalence, enhancing decision-making for researchers and policymakers.

Second, unlike model-specific clustering approaches, RaCE is broadly applicable across different NMA modeling frameworks, including frequentist and Bayesian methods, contrast-based and arm-based models, and various outcome types, such as binary, continuous, and survival data. The only information needed to apply the RaCE model is the mean and variance–covariance of the relative effects for the interventions (e.g., mean and variance–covariance matrix of the joint sampling distribution or posterior distribution of intervention effects). This flexibility allows researchers to apply RaCE to a wide range of NMA settings without being constrained by the underlying estimation model. By leveraging intervention effect estimates from any NMA model as input, RaCE ensures compatibility with evolving methodological advancements and accommodates different data structures. This adaptability makes it a valuable tool to facilitate diverse NMA applications.

Third, we develop RaCE for NMA by tailoring the rank-clustered BTL model,^20^ an advanced statistical model that naturally enables probabilistic grouping of interventions based on their relative effectiveness while accounting for ranking uncertainty. This approach does not require pre-identifying ranking structures among the interventions, such as the number of rank-clusters or overall ranking of interventions, or the commonly adopted minimal clinically important difference (MCID) to manually adjust the binary pairwise comparison and strict rank ordering. Instead, the RaCE model estimates similar interventions in a unified Bayesian approach, ensuring a more interpretable and practically relevant ranking structure. While RaCE also produces adjusted individual intervention effects, these values serve only as intermediate steps rather than direct interpretations, reinforcing the focus on meaningful rank-clusters rather than absolute ranks.

Fourth, RaCE is appropriately conservative when rank-clustering interventions based on their relative effects. As shown in simulation study 1 (Section 3.1), the model often assigns interventions with identical means and standard deviations in their relative effects a posterior probability of rank-clustering between 0.5 and 0.9. Although identical or near-identical estimates of relative effects may suggest perfect rank-clustering of interventions, uncertainty in the relative effects suggests that distinct effects are quite possible in reality. Thus, the model captures clustering among similar interventions without erroneously assuring equal intervention effects.

Fifth and last, RaCE infers accurate rank-clusters among interventions whose intervention effects are highly uncertain based on the result of a previous NMA study. In NMA, intervention rankings are influenced by the number of trials and network structure.^2^ For example, treatments with fewer trials tend to receive positively biased rankings, while extensively studied treatments may have negatively biased rankings.^3^ Consequently, this can lead to inaccurate rankings, favoring less-researched and inferior treatments over the well-established and causing seriously inflated false positive rates.^24^ Simulation Study 2 (Section 3.2) demonstrates that the RaCE model properly addresses this limitation by permitting extensively-studied and effective interventions to share the top rank, resulting in a much narrower RaCE credible interval when appropriate.

Despite its advantages, the proposed method has some limitations. RaCE counts heavily on the quality of NMA model estimates as its input data. For example, a common assumption in NMA modeling is evidence consistency, i.e., direct comparisons share the same overall effects as indirect comparisons. If this assumption does not hold in the NMA model, our rank-clustering result becomes unreliable. Therefore, RaCE users should carefully assess NMA model assumptions and select the most appropriate estimation approach for their specific application. Additionally, RaCE assumes normality in the distribution of effect estimates, which serves as the input for clustering. While this assumption is widely accepted and valid in most NMAs, deviations from normality could affect clustering accuracy. Future research should explore extensions that relax this assumption to accommodate more complex intervention effect distributions. Finally, the current method focuses on univariate outcomes, limiting its applicability in settings where multiple treatment endpoints (e.g., efficacy^21^ and safety^25^) must be considered simultaneously. Expanding RaCE to handle multivariate outcome clustering would further enhance its utility in clinical decision-making and policy development.

RaCE provides a flexible and interpretable solution to rank-clustering in NMA, enabling the identification of intervention groups with comparable effects. By decoupling rank-clustering from NMA modeling, it ensures broad applicability across different estimation frameworks and enhances the utility of NMA findings for policy decision-making. Overall, this method represents an important step toward more transparent and practical reporting of intervention rankings, aligning with the emerging needs of real-world applications.

## Figures and Tables

**Figure B1:**
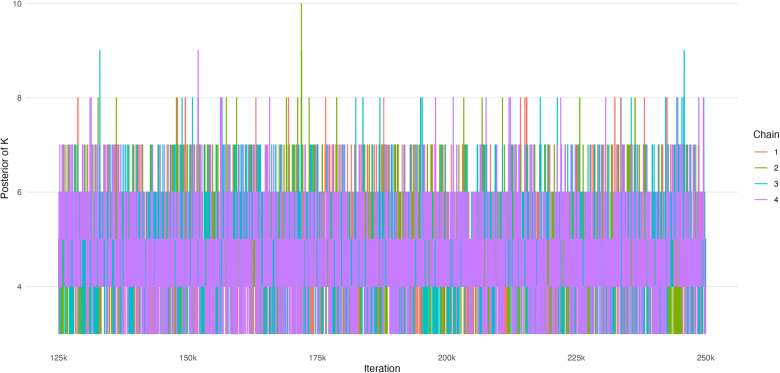
Trace plot of the number of rank-clusters, *K*.

**Figure B2:**
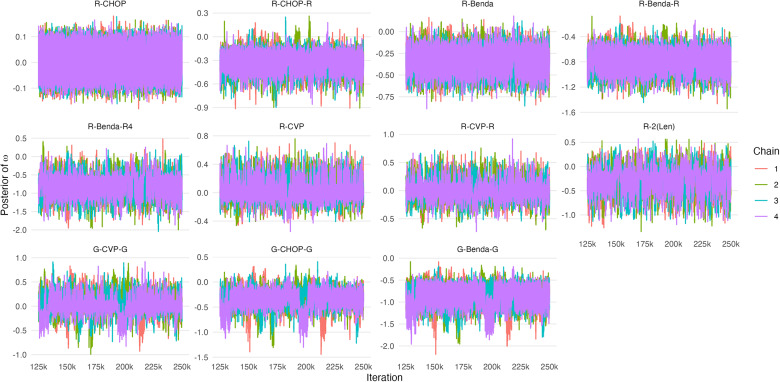
Trace plot of intervention-specific means, 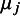 .

**Figure B3:**
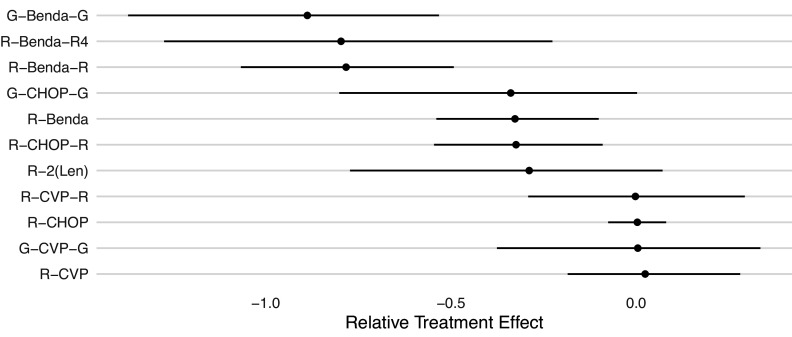
Forest plot of the posterior distributions of the relative treatment effects of 11 front-line immunochemotherapies for follicular lymphoma based on the proposed rank-clustering model. Point estimates and 95% credible intervals are shown. A smaller value indicates a more effective treatment.

## Data Availability

Code to implement the rank-clustered estimation (RaCE) model for network meta-analysis and replicate our analyses of real and simulated data may be found at https://github.com/pearce790/RaCE.NMA.

## Footnotes

1 Since there are more partitions with intermediate numbers of clusters than partitions with very few or many clusters, the uniform prior favors intermediate numbers of clusters *a priori*. However, we find the proposed model effectively identifies cluster structures (see Section 3).

2 In log-hazard ratio, with the lower value indicating a better treatment effect.

3 Rank-clusters may not always be as visually apparent as in this Case Study (see Figure 4). In such cases, one should analyze model results by direct interpretation of the posterior probabilities that each treatment belongs to each rank.

## A Details of Algorithm 1

*Algorithm 1—Step 2(a)*: In Step 2(a), we sample a new partition  conditional on the current partition *g*. Since  are intricately tied,  must simultaneously be updated to an appropriate  . We follow the seminal work of ^22^ on reversible-jump MCMC.

In each iteration, we propose  which are slight modifications of *g*: Precisely, we allow only for “births” splitting one cluster into two, or “deaths” merging two clusters into one. Since all partitions have positive probability, this process is irreducible, as required. There is no need to propose  that shuffle the partitions but maintain the number of clusters, as these partitions may be obtained by successive birth and death moves.

Births are attempted with probability  . In a birth, we select a cluster *k* at random among those with at least two objects. The cluster is split “binomially”, meaning that each object is placed independently into one of the “child” subgroups,  or  , with equal probability, conditional on each subgroup ultimately containing at least one object. Deaths are attempted with probability  . In a death, two adjacent clusters are merged at random. Adjacency means that  .

Births and deaths require updating  by increasing or decreasing its dimension by 1, respectively. In a birth, we split a cluster’s worth  into  using

(A.1)

where  .  should be chosen by the user based on the scale of the surrogate data. We suggest setting  , and adjusting higher (lower) if the Metropolis–Hastings acceptance probability is near 1 (near 0) in a trial run of the sampling algorithm. The corresponding death solves these equations simultaneously:

(A.2)

For reversibility, we automatically reject proposed births where  are not adjacent.

The Metropolis–Hastings probabilities for a birth and death, respectively, are  and  , where

(A.3)

where  is the transition probability of sampling  given current parameter set  .^22^

We now calculate each term in *A*. First,

(A.4)

where  is defined by Equation (2.1). Second,

(A.5)

The numerator in Equation (A.5) is the death probability times the probability of selecting a pair of adjacent partitions given  total partitions after a split (there are  such pairs). The denominator is the birth probability times the probability of selecting a specific cluster *k* among those with at least two members. This term also includes the probability of dividing the  objects in cluster *k* into two non-empty subsets. There are  such subsets, since there are  total possible partitions, two empty partitions, and two ways to obtain each two-way split. Third and last,

*Algorithm 1—Step 2(b)*:

Step 2(b) proceeds differently when the supplied covariance matrix  is diagonal or not. In the latter (standard) case, we update each  ,  , sequentially via a standard Metropolis–Hastings algorithm. In the former case, we notice that each  is assumed independent, thus permitting closed-form sampling of each  via its full conditional: Specifically, for every  ,

For ease of notation, let  , i.e., the product of the prior variance and the cluster’s object variances. Continuing on, we have

We note that the conjugate posterior derived above is distinct but similar in form to that of a standard Normal–Normal conjugate family, in that the posterior mean is a weighted average of the prior mean and data mean(s), with weights determined by the prior variance and data variance(s).

## B Further results from case study

First, we display trace plots of the number of rank-clusters, *K*, and the intervention-specific means,  . Figure B1 displays estimates of *K* across 4 chains of 250k iterations each (the first half were removed as burn-in). We observe good mixing and convergence among the chains. Figure B2 displays estimates of  across 4 chains of 250k iterations each, where again the first half were removed as burn-in. We observe good mixing and convergence among the chains.

Second, we conduct a posterior predictive check by examining estimates of the intervention-specific posterior distributions according to the RaCE model (Figure B3). We observe that the relative treatment effect is similar to that of Wang et al.,^21^ as replicated in Figure 5. There are two important differences. First, there is some shrinkage toward the mean of effect across all treatments, as is common in Bayesian models. Second, some the posterior distributions of some treatments shrink toward each other. This is the desired consequence of the rank-clustering model.
